# Analyses of carnivore microsatellites and their intimate association with tRNA-derived SINEs

**DOI:** 10.1186/1471-2164-7-269

**Published:** 2006-10-23

**Authors:** Francesc López-Giráldez, Olga Andrés, Xavier Domingo-Roura, Montserrat Bosch

**Affiliations:** 1 Genètica de la Conservació, Institut de Recerca i Tecnologia Agroalimentàries, Ctra. de Cabrils Km 2, 08348 Cabrils (Barcelona), Spain; 2 Deceased author

## Abstract

**Background:**

The popularity of microsatellites has greatly increased in the last decade on account of their many applications. However, little is currently understood about the factors that influence their genesis and distribution among and within species genomes. In this work, we analyzed carnivore microsatellite clones from GenBank to study their association with interspersed repeats and elucidate the role of the latter in microsatellite genesis and distribution.

**Results:**

We constructed a comprehensive carnivore microsatellite database comprising 1236 clones from GenBank. Thirty-three species of 11 out of 12 carnivore families were represented, although two distantly related species, the domestic dog and cat, were clearly overrepresented. Of these clones, 330 contained tRNA^Lys^-derived SINEs and 357 contained other interspersed repeats. Our rough estimates of tRNA SINE copies per haploid genome were much higher than published ones. Our results also revealed a distinct juxtaposition of AG and A-rich repeats and tRNA^Lys^-derived SINEs suggesting their coevolution. Both microsatellites arose repeatedly in two regions of the insterspersed repeat. Moreover, microsatellites associated with tRNA^Lys^-derived SINEs showed the highest complexity and less potential instability.

**Conclusion:**

Our results suggest that tRNA^Lys^-derived SINEs are a significant source for microsatellite generation in carnivores, especially for AG and A-rich repeat motifs. These observations indicate two modes of microsatellite generation: the expansion and variation of pre-existing tandem repeats and the conversion of sequences with high cryptic simplicity into a repeat array; mechanisms which are not specific to tRNA^Lys^-derived SINEs. Microsatellite and interspersed repeat coevolution could also explain different distribution of repeat types among and within species genomes.

Finally, due to their higher complexity and lower potential informative content of microsatellites associated with tRNA^Lys^-derived SINEs, we recommend avoiding their use as genetic markers.

## Background

Eukaryote genomes contain repetitive DNA sequences that can be classified into two groups: tandemly repeated sequences (e.g., micro- and minisatellites) and dispersed sequences (e.g., long interspersed elements – LINEs – and short interspersed elements – SINEs). Microsatellites (MSs) are tandem repeats of a DNA motif, one to six bases long, showing high levels of polymorphism based on changes in the repeat number. They are highly abundant and considered selectively neutral sequences and almost randomly distributed in the mammalian genome [1]. In spite of the wide applications of MSs and their importance in genetic and evolutionary studies, the mechanisms of the genesis of these sequences are still not fully understood. It is thought that point mutation is the dominant source of generation of short repeat MSs before slippage becomes the dominant mechanism [2-5]. However, based on the close association described between retroposons and MSs in several mammalian species (in sheep [6]; in pig [7]; in primates [8]; in humans [9]; in horse [10]), a completely different mechanism for MS generation has been proposed: it is thought that retrotranscripts undergo 3' polyadenylation – similar to mRNA polyadenylation [11] – prior to their incorporation into the genome, and that the extension of this preexisting repeat can generate A-rich MSs [8,9]. Nevertheless, this mechanism would not explain the recent observations (in barley, [12]; in Dipterans, [13]) that MSs can also be associated with both 5' and internal regions of some retroposons. An explanation for the latter association is the mechanism described by Wilder and Hollocher [13] which implies the conversion of an existing sequence with high cryptic simplicity located in Dipteran *mini-me *elements into tandemly repeated DNA. The importance of repeat elements as MS sources is still unclear. Whereas Nadir et al. [9] proposed Alu elements as the preferential source for the origin of human MSs, Morgante et al. [14] showed a significant association between MSs and the single/low-copy fraction of the plant genome.

**Figure 1:**
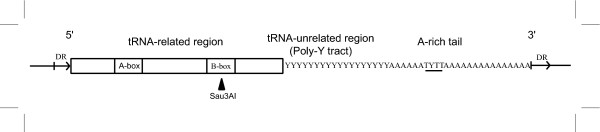
**Typical structure of a carnivore tRNA SINE**. Typical structure of a carnivore tRNA SINE with two promoter elements for RNA polymerase III (A-box and B-box), a polypyrimidine region and an A-rich tail with polymerase III termination signal (underlined). Direct repeats (DR) that result from the insertion process lie at both termini of the interspersed repeat. Restriction site for *Sau*3AI enzyme is indicated.

In mammals, most retroposons can be classified into three basic types: SINEs, LINEs, and LTR (Long Terminal Repeat) elements, with SINEs being the most abundant. SINEs are 80–400 bp long genomic repeats, apparently originating from tRNA (with the exception of human Alu and rodent B1 families which are derived from 7SL cytoplasmatic RNA, [15]). A typical SINE is flanked by short direct repeats and consists of three regions: a tRNA-related region, which contains an internal promoter for RNA polymerase III; a central family-specific or tRNA-unrelated region; and an A-rich tail (fig. 1; for a review of SINEs, see [16]). In carnivores, the tRNA-derived SINEs (tRNA SINEs), also known as CAN-SINEs, are thought to derive from tRNA^Lys ^[17]. They are also characterized by a polypyrimidine region (poly-Y) in their central region, and a polyadenylation AATAAA signal and the RNA polymerase III TTTT or TCTT terminator in the A-rich tail [17,18]. The tRNA-related region is more conserved than the unrelated part and the RNA polymerase III promoter is the most conserved, while poly-Y and A-rich tail are highly variable both in sequence and in length [17]. At first, it was thought that tRNA SINE distribution was limited to doglike carnivores (Canoidea superfamily; e.g.: dogs, bears, raccoons, weasels, skunks, and seals). However, recent works [17,18] have detected the tRNA SINE in the genome of several species of catlike carnivores (Feloidea superfamily; e.g.: hyenas, cats, mongooses, and civets) but not beyond carnivores.

In this study we constructed a comprehensive database of carnivore MS clones from GenBank to explore the role of interspersed repeats in the generation of repeat arrays. We focused on tRNA SINEs which constitute the best characterized and most abundant interspersed repeat element in the carnivore genome. We observed two modes of MS genesis in two regions of tRNA SINEs in which MSs have repeatedly evolved. This observation led us to hypothesize that SINEs generating MSs could explain part of the different repeat array content and distribution among and within genomes. We also demonstrated that MSs associated with tRNA SINEs were more complex and had less potential instability than those not associated. The observations reported here therefore have practical implications since the use of MSs related with tRNA SINEs calls for special attention from the designing of experiments to the interpretation of results.

## Results

### Database description

We obtained a non-redundant database of 1236 MS-containing clones for a total length of 515,359 bp representing 33 species from 11 of the 12 carnivore families (see table 1). The Mephitidae family was not included. However, there was a clear overrepresentation of domestic cat (*Felis catus*), contributing 40.8% of the total number of clones and 33.6% of total length, and domestic dog (*Canis familiaris*), with 15.0% and 23.7%, respectively. Each one of these two distantly related species is representative of the two major clades of carnivores: Feloidea and Canoidea superfamilies. The rest of the species contributed less than 8% of the two values, number of clones and sequence length.

### Identification and characterization of interspersed repeat elements

RepeatMasker masked 687 (55.5%) clones; of these, 330 contained tRNA SINEs, 292 LINEs, 93 LTR elements, 75 MIRs (mammalian-wide interspersed repeat), and 48 DNA transposons. It was possible for the same clone to contain more than one kind of interspersed repeat. In the 330 tRNA SINE-containing clones, we found 362 tRNA SINEs of which 47 were full-size. Thirty-two clones therefore contained two SINEs, in 27 of which they were oriented in the same way. We also explored the non-masked clone sequences using pairwise comparisons with BLAST to search for potentially new repeat elements but no new elements were found.

We obtained a rough estimate of the number of tRNA SINE copies per haploid genome for those species with the highest number of clones (table 1). We estimated 2.1*10^6 ^tRNA SINEs in cat, 9.1*10^5 ^in dog, 2.3*10^6 ^in badger, 2.0*10^6 ^in giant panda, and 4.5*10^6 ^in spotted hyena.

### Repeat motifs associated with interspersed repeat elements

A Sputnik search in our database for MSs revealed 1695 repeat arrays in the 1236 clone sequences. Table 2 shows the most abundant (found >5 times) repeat motifs. We identified 58 out of the 151 possible motifs from monomer to pentamer. The 72.1% of the repeat arrays were dinucleotides and 16.4% were tetranucleotides, while mono-, tri- and pentanucleotides each accounted for less than 5% of the total. None of the representatives of the (CRG)_n _MS family, which are mainly located either within or very close to coding sequences [19], was found. All tri-, tetra- and pentanucleotides have an A in their repeat unit.

When Sputnik was applied to the masked clones, we found that 557 and 454 out of the 1695 repeat arrays were associated with tRNA SINEs and other repeats, respectively. Out of 58 repeat motifs, 10 were not represented in the masked sequences, but this could be due to their low abundance in the whole database (each was found only 1 to 3 times).

### Implications of tRNA SINEs for the genesis of MSs

Overall, the abundance of motifs differs among databases (*P *< .0001; table 2). When pairwise comparisons were conducted, there were no significant differences in motif abundance between non-masked and other repeats clones (*P *= .0862) but there were significant differences between tRNA SINE and the other two databases (P values < .0001).

**Figure 2:**
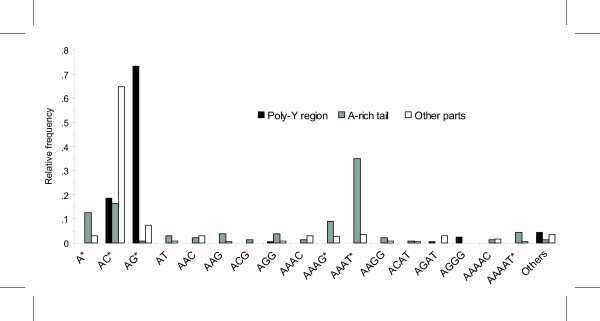
**Relative abundance of repeat motifs within tRNA SINE regions**. Relative abundance of repeat motifs within tRNA SINE regions: poly-Y (*N *= 161), A-rich tail (*N *= 134), and other parts (*N *= 227). Differences in specific motif abundance were tested using Fisher's exact tests comparing specific region/motif with the combined values of the other two regions. Repeat motif with frequencies which have a significant departure compared to Bonferroni-corrected alpha for 18 comparisons (*P*-value < .0028) are indicated with an asterisk (*). Thirty-five MSs were excluded because they were associated with SINEs which did not have a typical structure.

The tRNA SINE clones had a highly significant lack of AC repeats (22.5%, Fisher exact test, *P *< .0001) and an abundance of AG (65.9%, Fisher exact test, *P *< .0001) and AAAT (86.4%, Fisher exact test, *P *< .0001) repeats compared with the combined values of the other two databases (table 2). To know in detail how these differences were produced, we classified MSs in relation to the location in the tRNA SINE clones (fig. 1, fig. 2 and Supplementary Material table 1 [see Additional files 1]). Again, we found high significant differences in the overall motif distribution within the tRNA SINE clone sequence (*P *< .0001) and in pairwise comparisons among the different regions: poly-Y, A-rich tail and other parts (all three comparisons yielded a *P *< .0001). Poly-Y region had a highly significant abundance of AG repeats (86.8%, Fisher exact test, *P *< .0001) and the A-rich tail had a high number of A (70.8%, Fisher exact test, *P *< .0001), AAAG (66.7%, Fisher exact test, *P *= .0002), AAAT (85.5%, Fisher exact test, *P *< .0001), and AAAAT (85.7%, Fisher exact test, *P *< .0014) repeats. In addition, both regions showed a lack of AC repeats (15.1% and 11.1% respectively, Fisher exact test, *P*-values < .0001). No MSs were found in any other parts of the SINE sequences.

These results were based on the 330 tRNA SINE-containing clones but only 188 of these (15.1% of the total number of clones) had both poly-Y and A-rich tail (fig. 1). In the other clones, these regions were either not clearly distinguishable (*N *= 25) or there was only a fragment of the tRNA related region (*N *= 117). Taking into account only those clones containing SINEs with both regions, we observed that 65.0% of the poly-Y and 53.2% of the A-rich tail generated MSs. In these clones, 314 MSs were found to be distributed as follows: 51.3% in poly-Y, 42.7% in A-rich tail, and 6.1% downstream from the SINE. In a detailed analysis of the SINE sequence (fig. 2 and Supplementary Material table 1 [see Additional files 1]), the poly-Y tract produced 11 repeat motifs, which were mainly AG (73.3%) repeats. On the other hand, A-rich tail produced a higher motif diversity (17 repeat motifs), although these were limited to A-rich MSs. Thus, although only 188 clones had both regions, these differences in distribution of MSs among databases were mainly produced by poly-Y region which concentrated 55.1% of the AG and only 3.1% of AC repeats found in the whole database and by the A-rich tail which concentrated 71.2% of the AAAT and only 2.2% of AC repeats found in the whole database.

To discard that the observed significant tendencies were a result of the bias in species composition, we repeated the same statistical analyses with three different subsets of data: one containing only domestic dog clones, the second including cat clones, and the third compiling clones from other carnivore species. In all cases we observed the same tendencies corroborating that our findings are a common pattern in carnivores. The abundance of motifs among the databases and the statistics for these three subsets are shown in the Supplementary Material Table 2–4 [see Additional files 2, 3, 4]. We also repeated all the following analyses for these subsets.

### Implications of tRNA-SINEs for MS instability

**Figure 3:**
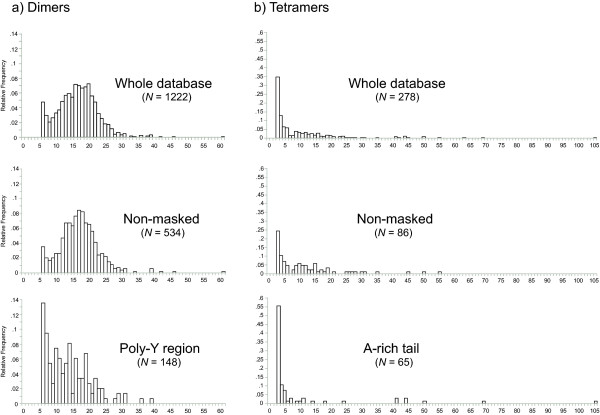
**Frequency distribution of repeat array length**. Relative frequency of repeat array length (number of repeats) for the most abundant motif classes – a) dimers and b) tetramers – in: the whole database, non-masked clones, poly-Y region and A-rich tail.

Although we could not measure MS polymorphism, repeat array length correlates well with MS instability. Overall, dimers and tetramers presented highly significant differences in size frequency distributions (fig. 3; Kolmogorov-Smirnov test, *N *= 1500, *Z *= 8.326, *P *< .0001). In general, the most common allele for dimer distribution was shifted further from the minimum MS size than tetramers (fig. 3). When comparing dimer distribution between MSs in non-masked clones and poly-Y region – which mainly produced dimers (see above) -, we observed that poly-Y region became more truncated and had shorter alleles (Fig 3). For tetramers, the shape of size frequency distribution is similar in both databases but A-rich tail – which mainly produced tetramers (see above) – had usually shorter alleles than non-masked clones (Fig. 3). Mean array length can be used to summarize the location of the frequency distribution, although this does not capture the entire spectrum of variation in these non-normal distributions (fig. 3; Kolmogorov-Smirnov test, Dimers *N *= 1222, *Z *= 1.938, *P *= .0011; Tetramer *N *= 278, *Z *= 4.736, *P *< .0001). On average for dimers, arrays in non-masked clones and poly-Y region had 17.0543 and 13.723 repeats, respectively. For this class, poly-Y region contained significantly shorter arrays than non-masked clones (Mann-Whitney U test; *N *= 682, *Z *= -6.240, *P *< .0001). On average for tetramers, arrays in non-masked clones and A-rich tail had 11.326 and 10.338 repeats, respectively. For this class, the A-rich tail generated significantly shorter arrays than non-masked clones (Mann-Whitney U test; *N *= 151, *Z *= -3.709, *P *= .0002). As we did for the other analyses, we also repeated these comparisons for the three subsets, except for tetramer within cats, which have a small sample size (*N *= 7). We obtained the same results with one exception (data not shown). We did not find significant differences when comparing tetramer MSs contained in non-masked clones and A-rich tail within dog but this is explained by an outlayer length value (Fig 3). When this value is removed from the analysis, differences become statistically significant (data not shown).

It is important to note that these comparisons do not represent assessments of orthologous loci, they represent one randomly chosen allele from each of many microsatellite loci within a species. Thus, the observed length variation among our tRNA-SINE clones may be due to variation among tRNA-SINE family or subfamily sequences. However, slippage occurs in MSs located in poly-Y and A-rich region, generating length variability within species after the insertion of the SINE. This can be observed in published tRNA-SINE clones containing a single variable MS in either poly-Y and A-rich tail (e.g. doglike: domestic dog [20], Eurasian badger (*Meles meles*) [21], American marten (*Martes Americana*), wolverine (*Gulo gulo*), and American badger (*Taxidea taxus*) [22]; catlike: spotted hyena (*Crocuta crocuta*) [23], Asiatic lion (*Panthera leo persica*) [24], and small Indian mongoose (*Herpestes javanicus*) [25]).

### Implications of tRNA-SINEs for MS complexity

To elucidate whether the contiguous existence of poly-Y and A-rich tail or similar structures in other interspersed repeats may be a source of compound MSs, we compared the presence of this kind of MSs among databases. Out of 550 non-masked clones, 66 contained compound arrays. The number of compounds was not significantly different from that calculated from the tRNA SINE (46 compounds in 330 clones, Fisher's exact test, *P *= .263) and other repeats (50 compounds in 357 clones, Fisher's exact test, *P *= .249) databases. Even when we calculated the number of compounds in the tRNA SINEs database taking into account only clones with both poly-Y tract and A-rich tail, there were no significant differences between databases (data not shown). Moreover, we did not find any significant increase in the number of compound MSs in the SINE database when we changed the number of non-repeat nucleotides separating two adjacent arrays (data not shown). However, the presence of these two regions (poly-Y and A-rich tail) should imply a source of multiple arrays in the same clone. The number of clones with multiple arrays in tRNA SINE sequences was 146 which was significantly higher than the number calculated for non-masked sequences (128 multiples in 550 clones, Fisher's exact test, *P *< .0001) and for other repeats (85 multiples in 357 clones, Fisher's exact test, *P *< .0001).

On the other hand, of the 684 repeat arrays in non-masked clones, 181 were imperfect and this number was not significantly different from the number calculated for tRNA SINEs (156 imperfects, Fisher's exact test, *P *= .2928) and for other repeats (115 imperfects, Fisher's exact test, *P *= .3613).

The same tendencies were apparent when the three species subsets were analysed separately (data not shown).

## Discussion

Our database was generated from MS-containing clones of carnivore species which are generally used in population genetics and individual identification studies. These MSs had been isolated following the traditional method, which is known to produce a nonrepresentative sample of the genome. Two common procedures repeatedly used in traditional MS isolation that may cause biases in our database are: i) the use of *Sau*3AI restriction enzyme to fragment genomic DNA, and ii) the use of AC probes to screen the libraries. However, and despite the biases inherent in isolation methods, the drawn conclusions are still pertinent or in some cases even reinforced (see below).

*Sau*3AI recognizes sites that are well-conserved in dispersed repeats (e.g., in porcine PRE-1, in rat L1 elements), and in the B-box of the tRNA polymerase III promoter of our tRNA SINEs. As a result, the SINEs found in our databases were often truncated (87.0%), conserving the 5' end in 127 cases and the 3' end, including the poly-Y and A-rich tail regions, in 188 cases. We found 47 full tRNA SINEs which could be used as phylogenetic markers. This fact has led some authors (e.g. [26,27]) to state that the use of *Sau*3AI may result in bias towards the isolation of repeat-associated MSs. However, such a bias could not exist taking into account the high frequency of cutting owing to the restriction recognition site for *Sau*3AI (^GATC) is only four nucleotides in length.

The preferential use of (AC)_n _probes for library screening was reflected in the repeat motif content of our database where 58% were AC repeats, clearly overrepresented [28]. However, in this work we have demonstrated that AC repeats are not statistically associated with tRNA-SINEs – only 5.3% of the AC repeats in the whole database were located within the SINE sequence. Similar results have been found in humans where more than 80% of AC repeats are not associated with Alu sequences [8,9,29]. Using AC probes would therefore reduce the final number of SINEs captured in the MS isolation process.

Our tRNA SINE copy estimates per haploid genome in dog (9.1*10^5^) were very similar to the values obtained from the dog genome sequence (1.06*10^6 ^[30]). It seems then reasonable to think that the values we obtained for the other carnivore species (2.1*10^6 ^tRNA SINEs in cat, 2.3*10^6 ^in badger, 2.0*10^6 ^in giant panda, and 4.5*10^6 ^in spotted hyena) may also be realistic approximations. Even in the case where the above mentioned biases do apply – probably reducing tRNA SINE captured -, it is unlikely they would produce a difference of an order of magnitude with published estimates (10^5 ^– 3*10^5 ^in mustelids, 2*10^5 ^in cats and true seals, and 1.5*10^5 ^– 4*10^5 ^in dogs and bears [17,26,31,32]).

We also observed that poly-Y (65.0%) and A-rich tail (53.2%) regularly gave rise to MSs. The different repeat motifs derived from these regions (mostly AG and AAAT, respectively) suggested two mechanisms for MS generation. The first mechanism was illustrated by the A-rich tail and it has already been well described in human Alu sequences [8,9]. This mode of genesis implies the presence of a pre-existing MS and subsequent modifications by point mutation and slippage events. It has been suggested that the pre-existing MS could arise from the incorporation of the retrotranscript with an extended polyadenylated tail, a feature which may also serve to guide their retroposition in the genome [9]. Although the pre-existing MS was an adenine tract, this evolved into more complex structures where we observe certain variability in the repeat motif, mostly centered in A rich MS (A 12.6% and A_2–4_N 57.8%).

The second mechanism is based on the fact that the poly-Y region has a nucleotide composition highly biased towards pyrimidines. It could therefore be defined as a site with high cryptic simplicity [33], which is a DNA sequence biased in nucleotide composition and made up of short sequence motifs that, initially, are not tandemly repeated. The functional significance of this structure currently remains unknown, but its presence in these elements makes the SINE an important source of MS genesis. Generation of MSs at this site depends on base substitutions that create a tandemly duplicated motif, and on subsequent slippage mutations to increase the number of copies. Thus, a few C ↔ T transitions, the most frequent substitutions, are enough to transform cryptic simplicity sequences into tandemly repeated DNA. The initial bias in the base substitution was reflected in the repeat motifs generated, 73.4% of which were AGs. This tendency for invariability and the greater number of MSs produced by the poly-Y region suggest that slippage mutation is active during the early stages of MS genesis. This MS-generating mutation process has also been shown in an internal region of the mini-me elements of Dipterans [13]. The poly-Y region has also been found to be specific of other mammalian SINEs, such as rabbit C repeat [34], rodent DIP [35], bat VES [36], and insectivore TAL, ERI-1 and ERI-2 [37], showing that the action of the two mechanisms that generate MSs are not exclusive to the tRNA SINEs.

It is known that different MS motifs, motif classes and even abundances are not equally represented in species belonging to different groups [28,38] or even within the genome of any one particular species [39,40]. These differences are still not well-understood and it has been hypothesized that they may be caused by species-specific differences in the DNA synthesis and repair machinery [41], selection [42], or base composition [43]. Although tRNA SINEs are not the only source of MSs in carnivore genomes, these elements could explain part of the differences in the distribution of MSs within a particular genome due to their high abundance and their preference for insertion at specific sites – such as around the R bands [44,45] and clustering or insertion into other mobile genetic elements [9,26,46]. They could also explain differences among phylogenetically distant species or groups, since the interspersed repeat families generating MSs may be lineage specific. Along these lines, our results would indicate that tRNA SINEs have a significant effect on the overall distribution of some repeat motifs in carnivores, especially AG and AAAT repeats.

The strong association between repeat elements and MSs has been largely used for different purposes, such as: i) to develop new, codominant multiplex marker technologies such as S-SAP [47] or inter-AluPCR [48]; ii) to build MS-enriched libraries by amplifying *Sau*3AI inserts with a conserved SINE primer and a flanking vector primer [49]; iii) to discover new SINEs, especially in species for which little information is available concerning their repeat element content [50]; and iv) to discover new SINE loci which could be used to reconstruct phylogenies [51]. In this study we detected 47 completed SINEs whose flanking regions are targets for primer design and could be used as phylogenetic markers.

In spite of these applications, MSs associated with interspersed repeats not only distort estimates of the genomic distribution of MSs useful for genome mapping [27], but also entail some methodological disadvantages. Firstly, genotyping with MSs associated with repeat elements is very hard. Placing one of the PCR primers within a highly repeated element might cause weak amplification, high background and difficulty in locus-specific amplification [10,27,52]. Moreover, if the primers were designed up- and downstream from the repeated element, the expected large size of the PCR product might cause problems in the resolution of the amplified products [53]. Our results also showed that potential instability in MSs associated with tRNA SINEs was lower than in non-associated MSs. Several studies (e.g. [54-56]) have shown that MS mutation rate increases with an increasing number of repeat units; this is considered the single most important factor affecting the mutation rate. The isolation of non-masked MS clones is therefore advisable on account of their high potential informative content. It has been argued that point mutations break up perfect repeats and reduce the mutation rates of MSs [57]. Since there are not significant differences in the number of imperfect repeat arrays among databases, the higher content of short repeat arrays in MSs associated with tRNA SINE cannot entirely be attributed to imperfections. Finally, most of the applications involving MSs as genetic markers are based on variations in the length of the PCR product, which is expected to vary according to single-step changes in the number of repeats. However, it has been shown that poly-Y is responsible for variation in length in the MS flanking region within species (López-Giráldez et al., unpublished data). This is probably due to the fact that cryptically simple sequences are susceptible to undergo slippage in a similar manner to MSs, but at lower rates [58]. We also detected a larger number of clones with multiple MSs in the tRNA SINE database owing to the presence of poly-Y and A-rich tail. This may also explain the non-neutral observation of MS clustering [59]. Both cases mentioned preclude the basic assumptions of MS mutational models. As a result, the interpretation of the data obtained from MSs associated with tRNA SINEs may induce erroneous conclusions. Thus, we propose avoiding the use of MS associated with interspersed repeats as genetic markers.

## Conclusion

In this report we have shown how tRNA SINEs, the most abundant carnivore and a lineage-specific SINE, are clearly responsible for generating an important fraction of carnivore MSs. More specifically, we have demonstrated that not only the A-rich tails but also an internal region (poly-Y) of these elements regularly expand into lengthy MSs via two different mechanisms: the expansion of pre-existing tandem repeats and the conversion of sequences with high cryptic simplicity into tandemly repetitive DNA. The MS genesis in tRNA-SINEs is not only involved in complex patterns, such as multiple repeated arrays and length variation in the flanking regions, and is responsible for shorter repeat arrays, but may also explain differences in MS distribution among and within species genomes. The mechanism we have described in tRNA SINEs may also be generalized to other interspersed repeats. Based on the negative effect of the association between MSs and interspersed repeats, we recommend avoiding the use of these MSs as genetic markers. We suggest applying computer tools after initial sequencing in order to detect interspersed repeats in MS-containing clones and taking special attention when designing isolation methods (e.g., not using *Sau*3AI and AG or AAAT probes).

## Methods

### Construction of a sequence database of MS-containing clones

We constructed a non-redundant database of clone sequences of carnivore MSs obtained from GenBank (Release 146.0). Firstly, we performed an *Entrez *[60] MS search limited to Genomic DNA and to carnivores. We also arbitrary limited our sequences to a minimum length of 200 bp to ensure the possibility of tRNA SINE detection and to obtain a reasonably sized dataset of identified MSs for analysis. We removed clone sequences which were not obtained following the traditional method of MS isolation – i.e. isolation from partial genomic libraries (selected for small insert size) or from MS-enriched libraries of the species of interest, and screening several thousands of clones through colony hybridization with repeat-containing probes [61]. To ensure this, we checked all MS publications and, for unpublished entries, we asked the authors to provide information concerning their MS isolation procedures. Secondly, we used the CLEANUP program [62,63] to detect and eliminate duplicated sequences and to facilitate the identification of different alleles of the same locus, as well as loci shared among species. Finally, we used the NCBI tool VecScreen [64] to eliminate possible vector contamination. Sequences which were reduced to below 200 bp after removing vector fragments were deleted from the database.

### Computer screening of the database for clones carrying interspersed repeats

We used the RepeatMasker program (Smit, Hubley, and Green, unpublished data; RepeatMasker open-3.0.8 at [65]) to detect and classify interspersed repeats in the clone database. To do this, RepeatMasker makes use of Repbase Update [66] which is a comprehensive database of repetitive element consensus sequences. We ran RepeatMasker at low speed using the interspersed repeat carnivore library as DNA source. In order to find possible repetitive DNA sequences not included in Repbase Update, the clones in which RepeatMasker did not find interspersed repeat sequences were analyzed by pairwise comparisons with the Basic Local Alignment Search Tool (BLAST, [67] downloaded from NCBI BLAST ftp site [68]).

To compare the repeat array content and evaluate the association between MSs and interspersed repeats, we further subdivided MS-containing clones into three different databases based on the RepeatMasker output: i) non-masked clones – i.e. not containing interspersed repeats; ii) clones masked as tRNA^Lys^-derived SINEs; and iii) the remaining masked clones- i.e. intimately associated with other interspersed repeats.

We roughly estimated the number of tRNA SINE copies for different carnivore species from the proportion of that element in bank sequences following the equation used by Bentolila et al. [26]: *N *= *n *× 3 × 10^9^/*L*; where *n *is the number of tRNA SINEs found in a species with a total length (L) represented in the database, and 3 × 10^9 ^states for the haploid length of a mammalian genome.

### Computer identification and characterization of repeat arrays

To identify all repeat arrays in the MS-containing clones following a standard criterion, we used the modified version of the Sputnik program (Abajian, unpublished [69]) used in Morgante, Hanafey, and Powell [14]. We looked for motifs of 1 to 5 bases repeated at least three times and with a total length of at least 12 bases. We allowed up to 10% variation between MS and a perfectly repeated motif of the same length (designated as imperfect and perfect MSs, respectively). We also considered a compound MS when two consecutive repeats detected by Sputnik were separated by no more than three consecutive non-repeat bases [70]. In all analyses, each repeat array of compound MSs was treated as an independent unit, unless comparison of compound MSs among databases was performed, in which case, the whole compound repeat was considered as a single array. Classification of MS sequences was carried out according to their repeat unit outputted by Sputnik, including all permutations on both strands (e.g., AAG represents the following: AAG, AGA, GAA, CTT, CTC, and TTC). Thus, the total number of theoretically possible repeat units is 151. Since two regions of tRNA SINEs have been associated with MSs [17], we classified the MSs associated with tRNA SINEs into three subtypes whenever possible: i) those which were positioned 3' or 5' from a transposable element; ii) those which had arisen at an internal sequence (poly-Y) and thus had transposable element sequences on both flanks; and iii) those which were part of the A-rich tail (fig. 1).

### Statistical analyses

To evaluate the consistency with which repeat motifs were represented among databases – and to overcome the problem of low cell counts -, we used a Monte Carlo approximation (*N *= 100,000) of Fisher's exact test. If differences were observed, we then used Fisher's exact tests to compare differences in specific motifs. Significance levels were adjusted using the standard Bonferroni method to take into account multiple tests on the same data set. To compare the length of the repeat array between different databases, we performed a Mann-Whitney U-test, after first conducting a normality test. To investigate whether complex MS structures (e.g., compound and imperfect MSs) were associated with the presence of interspersed repeats, we compared the abundance of complex MSs found in the different databases using Fisher's exact test. All statistical analyses were performed with SPSS v11.0.1 (SPSS Inc.).

## Authors' contributions

FL-G conceived and participated in the design of the study, participated in the database construction, wrote Perl scripts for sequence manipulation, performed the sequence and statistical analyses and drafted the manuscript. OA participated in database construction and sequences analyses and helped in the draft of the manuscript. XD-R coordinated and helped in the draft the manuscript. MB participated in the design of the study and database construction and coordinated and helped in the draft of the manuscript. All authors read and approved the manuscript.

## Supplementary Material

Additional file 1 **Supplementary Material Table 1**. Distribution of MSs associated with tRNA SINEs. Click here for file

Additional file 2 **Supplementary Material Table 2**. Distribution of the most abundant MSs in domestic dog for the different databases. Click here for file

Additional file 3 **Supplementary Material Table 3**. Distribution of the most abundant MSs in domestic cat for the different databases. Click here for file

Additional file 4 **Supplementary Material Table 4**. Distribution of the most abundant MSs in other carnivore species for the different databases. Click here for file

## Figures and Tables

**Table 1:** Distribution of MS-containing clones by species.

**Species**	**Family**	**Clones**	**bp**	**SINEs**	**Other repeats**	**Articles**	**Journals**
*Felis catus*	Felidae	504(40.8%)	173240(33.6%)	125	130	3	2,8,11,17
*Canis familiaris*	Canidae	185(15.0%)	122264(23.7%)	37	67	19	2,6,10,12,14,17
*Meles meles*	Mustelidae	82(6.6%)	33008(6.4%)	25	29	3	2,15
*Ailuropoda melanoleuca*	Ursidae	80(6.5%)	40181(7.8%)	27	33	2	1,5,17
*Crocuta crocuta*	Hyaenidae	75(6.1%)	21993(4.3%)	33	10	3	3,15,16,17
*Mustela vison*	Mustelidae	46(3.7%)	21642(4.2%)	11	14	3	4,15,17
*Panthera leo*	Felidae	30(2.4%)	12733(2.5%)	15	2	1	16,17
*Lutra lutra*	Mustelidae	21(1.7%)	8652(1.7%)	3	9	3	7,15,16,17
*Odobenus rosmarus*	Odobenidae	14(1.1%)	9232(1.8%)	1	5	1	15
*Zalophus californianus*	Otariidae	14(1.1%)	8027(1.6%)	2	6	1	16,17
*Herpestes javanicus*	Herpestidae	13(1.1%)	3725(0.7%)	7	1	1	16
*Ursus arctos*	Ursidae	13(1.1%)	5201(1.0%)	0	6	1	15
*Panthera tigris*	Felidae	12(1.0%)	3114(0.6%)	6	2	1	16,17
*Mungos mungo*	Herpestidae	11(0.9%)	3791(0.7%)	1	1	1	16
*Martes americana*	Mustelidae	11(0.9%)	2979(0.6%)	6	2	1	15
*Halichoerus grypus*	Phocidae	11(0.9%)	2822(0.5%)	0	5	2	12,15
*Ursus americanus*	Ursidae	11(0.9%)	3607(0.7%)	0	6	2	15,17
*Leptonychotes weddellii*	Phocidae	10(0.8%)	3257(0.6%)	4	0	2	13,16
*Ailurus fulgens*	Procyonidae	10(0.8%)	3395(0.7%)	2	3	1	16
*Lontra canadensis*	Mustelidae	9(0.7%)	3825(0.7%)	4	1	1	16
*Suricata suricatta*	Herpestidae	9(0.7%)	4971(1.0%)	2	3	1	16
*Mustela erminea*	Mustelidae	8(0.6%)	2514(0.5%)	1	4	2	12,15
*Ursus thibetanus*	Ursidae	8(0.6%)	3460(0.7%)	2	5	1	15
*Phoca vitulina*	Phocidae	7(0.6%)	2099(0.4%)	1	4	3	2,15
*Fossa fossana*	Viverridae	6(0.5%)	1585(0.3%)	1	1	1	16
*Gulo gulo*	Mustelidae	6(0.5%)	1337(0.3%)	1	0	2	15
*Urocyon cinereoargenteus*	Canidae	6(0.5%)	3291(0.6%)	0	3	1	16
*Alopex lagopus*	Canidae	5(0.4%)	2039(0.4%)	0	3	1	9,14
*Cryptoprocta ferox*	Viverridae	5(0.4%)	2388(0.5%)	4	0	1	15
*Hydrurga leptonyx*	Phocidae	4(0.3%)	1541(0.3%)	4	0	2	13,16
*Lobodon carcinophagus*	Phocidae	4(0.3%)	1335(0.3%)	1	1	2	13,16
*Lynx canadensis*	Felidae	3(0.2%)	1254(0.2%)	2	0	1	15
*Taxidea taxus*	Mustelidae	3(0.2%)	857(0.2%)	2	1	1	15
							
TOTALS		1236	515359	330	357	71	

SINEs: the number of clones associated with tRNA SINEs. Other repeats: the number of clones associated with other interspersed repeats. Articles: the number of publications in which these clones appeared. Journals: 1, Acta Genetica Sinica (*N *= 1); 2, Animal Genetics (*N *= 19); 3, Behavioural Ecology (*N *= 1); 4, Canadian Journal of Animal Science (*N *= 1); 5, Chinese Journal of Applied and Environmental Biology (*N *= 1); 6, Chromosome Research (*N *= 1); 7, Conservation Genetics (*N *= 1); 8, Cytogenetics and Genome Research (*N *= 1); 9, Electrophoresis (*N *= 1); 10, Genetics (*N *= 1); 11, Genomics (*N *= 1); 12, Journal of Heredity (*N *= 3); 13, Journal of Mammalogy (*N *= 1); 14, Mammalian Genome (*N *= 2); 15, Molecular Ecology (*N *= 16); 16, Molecular Ecology Notes (*N *= 15); and 17, Unpublished; where *N *shows the number of publications in each journal.

**Table 2:** Distribution of the most abundant MSs in the different databases.

Unit	Non-masked	tRNA SINEs	Other repeats	Total	*P*-value^a^
A	14	24	11	49	0.0179
C	4	0	4	8	0.0590
					
AC	476	221*	285	982	<.0001
AG	45	141*	28	214	<.0001
AT	7	6	5	18	>.9999
CG	6	0	2	8	0.0590
					
AAC	5	11	11	27	0.4108
AAG	2	6	2	10	0.0896
ACC	4	0	5	9	0.0349
AGC	6	0	2	8	0.0590
AGG	4	8	0	12	0.0250
					
AAAC	6	9	2	17	0.1157
AAAG	19	19	16	54	0.7686
AAAT	3	57*	6	66	<.0001
AAGG	13	5	16	34	0.0256
ACAG	4	0	5	9	0.0349
ACAT	2	3	2	7	0.6901
ACGC	4	0	2	6	0.1863
AGAT	13	9	19	41	0.1772
AGGG	4	4	0	8	0.4509
ATCC	8	0	3	11	0.0201
					
AAAAC	6	6	1	13	0.3747
AAAAT	1	6	1	8	0.0180
ACACC	3	0	3	6	0.1863
					
Others	25	22	23	70	0.8968
					
Total	684	557	454	1695	

^a^Fisher's exact test for comparisons between specific motifs in tRNA SINE and the combined values of the other two databases. Repeat motif frequencies which have a significant departure compared to Bonferroni-corrected alpha for 25 comparisons (*P*-value < .002) are indicated with an asterisk (*).
