# Supplementary material for: Analyses of carnivore microsatellites and their intimate association with tRNA-derived SINEs
Source: BMC Genomics. 2006 Oct 23;7:269. doi: 10.1186/1471-2164-7-269 (PMC1634856; doi:10.1186/1471-2164-7-269)
Supplement: Additional file 1 — Supplementary Material Table 1. Distribution of MSs associated with tRNA SINEs. [file 1471-2164-7-269-S1.doc]

**Supplementary Material Table 1.**

**Distribution of MSs Associated with tRNA SINEs.**

Fisher’s exact test for comparisons between specific poly-Y^a^, A-rich tail^b^ or other parts^c^ motifs and the combined values of the other two regions. Repeat motif frequencies which have a significant departure compared to Bonferroni-corrected alpha for 18 comparisons (*P*-value < .0028) are indicated *. ^d^Thirty-five MSs were excluded because they were associated with SINEs which did not have a typical structure.

| **Unit** | **Poly-Y region** | **A-rich tail** | **Other parts** | **Total** | ***P*-value^a^** | ***P*-value^b^** | ***P*-value^c^** |
| --- | --- | --- | --- | --- | --- | --- | --- |
| A | 0* | 17* | 7 | 24 | 0.0002 | <.0001 | 0.2054 |
|  |  |  |  |  |  |  |  |
| AC | 30* | 22* | 147* | 199 | <.0001 | <.0001 | <.0001 |
| AG | 118* | 1* | 17* | 136 | <.0001 | <.0001 | <.0001 |
| AT | 0 | 4 | 2 | 6 | 0.1842 | 0.0403 | 0.7015 |
|  |  |  |  |  |  |  |  |
| AAC | 0 | 3 | 7 | 10 | 0.0358 | 0.7219 | 0.1112 |
| AAG | 0 | 5 | 1 | 6 | 0.1842 | 0.0050 | 0.2399 |
| ACG | 0 | 2 | 0 | 2 | >.9999 | 0.0655 | 0.5075 |
| AGG | 1 | 5 | 2 | 8 | 0.4450 | 0.0295 | 0.4757 |
|  |  |  |  |  |  |  |  |
| AAAC | 0 | 2 | 7 | 9 | 0.0630 | >.9999 | 0.0451 |
| AAAG | 0* | 12* | 6 | 18 | 0.0014 | 0.0002 | 0.4715 |
| AAAT | 0* | 47* | 8* | 55 | <.0001 | <.0001 | <.0001 |
| AAGG | 0 | 3 | 2 | 5 | 0.3302 | 0.1097 | >.9999 |
| ACAT | 0 | 1 | 1 | 2 | >.9999 | 0.4479 | >.9999 |
| AGAT | 1 | 0 | 7 | 8 | 0.4450 | 0.1209 | 0.0240 |
| AGGG | 4 | 0 | 0 | 4 | 0.0088 | 0.5767 | 0.1363 |
|  |  |  |  |  |  |  |  |
| AAAAC | 0 | 2 | 4 | 6 | 0.1842 | 0.6495 | 0.4110 |
| AAAAT | 0 | 6* | 1 | 7 | 0.1058 | 0.0014 | 0.1448 |
|  |  |  |  |  |  |  |  |
| Others | 7 | 2 | 8 | 17 | 0.4232 | 0.2611 | 0.8069 |
|  |  |  |  |  |  |  |  |
| Total | 161 | 134 | 227 | 522^d^ |  |  |  |
