# Supplementary material for: Analyses of carnivore microsatellites and their intimate association with tRNA-derived SINEs
Source: BMC Genomics. 2006 Oct 23;7:269. doi: 10.1186/1471-2164-7-269 (PMC1634856; doi:10.1186/1471-2164-7-269)
Supplement: Additional file 2 — Supplementary Material Table 2. Distribution of the most abundant MSs in domestic dog for the different databases. [file 1471-2164-7-269-S2.doc]

**Supplementary Material Table 2**

**Distribution of the most abundant MSs in domestic dog for the different databases.**

^a^Fisher’s exact test for comparisons between specific motifs in tRNA SINE and the combined values of the other two databases. Repeat motif frequencies which have a significant departure compared to Bonferroni-corrected alpha for 25 comparisons (*P*-value < .002) are indicated with an asterisk (*).

| **Unit** | **Non-masked** | **tRNA SINEs** | **Other repeats** | **Total** | ***P-* value^a^** |
| --- | --- | --- | --- | --- | --- |
| A | 5 | 3 | 6 | 14 | 0.561 |
| C | 1 | 0 | 2 | 3 | 0.555 |
|  |  |  |  |  |  |
| AC | 48 | 10* | 27 | 85 | <.0001 |
| AG | 4 | 18* | 4 | 26 | <.0001 |
| AT | 0 | 1 | 1 | 2 | 0.519 |
| CG | 0 | 0 | 0 | 0 |  |
|  |  |  |  |  |  |
| AAC | 1 | 1 | 3 | 5 | 1.000 |
| AAG | 2 | 1 | 0 | 3 | 1.000 |
| ACC | 2 | 0 | 4 | 6 | 0.182 |
| AGC | 0 | 0 | 1 | 1 | 1.000 |
| AGG | 4 | 0 | 0 | 4 | 0.316 |
|  |  |  |  |  |  |
| AAAC | 1 | 2 | 0 | 3 | 0.222 |
| AAAG | 5 | 11 | 6 | 22 | 0.051 |
| AAAT | 1 | 19* | 1 | 21 | <.0001 |
| AAGG | 6 | 3 | 7 | 16 | 0.404 |
| ACAG | 0 | 0 | 1 | 1 | 1.000 |
| ACAT | 0 | 0 | 0 | 0 |  |
| ACGC | 0 | 0 | 0 | 0 |  |
| AGAT | 2 | 1 | 2 | 5 | 1.000 |
| AGGG | 1 | 0 | 0 | 1 | 1.000 |
| ATCC | 4 | 0 | 3 | 7 | 0.104 |
|  |  |  |  |  |  |
| AAAAC | 0 | 0 | 0 | 0 |  |
| AAAAT | 0 | 3 | 1 | 4 | 0.086 |
| ACACC | 2 | 0 | 0 | 2 | 1.000 |
|  |  |  |  |  |  |
| Others | 7 | 4 | 10 | 21 | 0.324 |
|  |  |  |  |  |  |
| Total | 96 | 77 | 79 | 252 |  |
