# Supplementary material for: Analyses of carnivore microsatellites and their intimate association with tRNA-derived SINEs
Source: BMC Genomics. 2006 Oct 23;7:269. doi: 10.1186/1471-2164-7-269 (PMC1634856; doi:10.1186/1471-2164-7-269)
Supplement: Additional file 3 — Supplementary Material Table 3. Distribution of the most abundant MSs in domestic cat for the different databases. [file 1471-2164-7-269-S3.doc]

**Supplementary Material Table 3**

**Distribution of the most abundant MSs in domestic cat for the different databases.**

^a^Fisher’s exact test for comparisons between specific motifs in tRNA SINE and the combined values of the other two databases. Repeat motif frequencies which have a significant departure compared to Bonferroni-corrected alpha for 25 comparisons (*P*-value < .002) are indicated with an asterisk (*).

| **Unit** | **Non-masked** | **tRNA SINEs** | **Other repeats** | **Total** | ***P-* value^a^** |
| --- | --- | --- | --- | --- | --- |
| A | 0 | 1 | 1 | 2 | 0.493 |
| C | 1 | 0 | 0 | 1 | 1.000 |
|  |  |  |  |  |  |
| AC | 251 | 124* | 130 | 505 | <.0001 |
| AG | 13 | 35* | 8 | 56 | <.0001 |
| AT | 1 | 2 | 0 | 3 | 0.201 |
| CG | 4 | 0 | 1 | 5 | 0.329 |
|  |  |  |  |  |  |
| AAC | 2 | 1 | 0 | 3 | 1.000 |
| AAG | 0 | 0 | 0 | 0 |  |
| ACC | 0 | 0 | 0 | 0 |  |
| AGC | 0 | 0 | 0 | 0 |  |
| AGG | 0 | 0 | 0 | 0 |  |
|  |  |  |  |  |  |
| AAAC | 0 | 2 | 0 | 2 | 0.083 |
| AAAG | 1 | 0 | 0 | 1 | 1.000 |
| AAAT | 1 | 5 | 1 | 7 | 0.023 |
| AAGG | 0 | 0 | 2 | 2 | 1.000 |
| ACAG | 1 | 0 | 2 | 3 | 0.561 |
| ACAT | 1 | 2 | 1 | 4 | 0.327 |
| ACGC | 2 | 0 | 2 | 4 | 0.583 |
| AGAT | 2 | 1 | 2 | 5 | 1.000 |
| AGGG | 0 | 1 | 0 | 1 | 0.288 |
| ATCC | 1 | 0 | 0 | 1 | 1.000 |
|  |  |  |  |  |  |
| AAAAC | 3 | 2 | 0 | 5 | 0.629 |
| AAAAT | 1 | 1 | 0 | 2 | 0.493 |
| ACACC | 1 | 0 | 2 | 3 | 0.561 |
|  |  |  |  |  |  |
| Others | 8 | 5 | 4 | 17 | 1.000 |
|  |  |  |  |  |  |
| Total | 294 | 182 | 156 |  |  |
