# Supplementary material for: Analyses of carnivore microsatellites and their intimate association with tRNA-derived SINEs
Source: BMC Genomics. 2006 Oct 23;7:269. doi: 10.1186/1471-2164-7-269 (PMC1634856; doi:10.1186/1471-2164-7-269)
Supplement: Additional file 4 — Supplementary Material Table 4. Distribution of the most abundant MSs in other carnivore species for the different databases. [file 1471-2164-7-269-S4.doc]

**Supplementary Material Table 4**

**Distribution of the most abundant MSs in other carnivore species for the different databases.**

^a^Fisher’s exact test for comparisons between specific motifs in tRNA SINE and the combined values of the other two databases. Repeat motif frequencies which have a significant departure compared to Bonferroni-corrected alpha for 25 comparisons (*P*-value < .002) are indicated with an asterisk (*).

| **Unit** | **Non-masked** | **tRNA SINEs** | **Other repeats** | **Total** | ***P-* value^a^** |
| --- | --- | --- | --- | --- | --- |
| A | 9 | 20 | 4 | 33 | 0.005 |
| C | 2 | 0 | 2 | 4 | 0.303 |
|  |  |  |  |  |  |
| AC | 177 | 87* | 128 | 392 | <.0001 |
| AG | 28 | 88* | 16 | 132 | <.0001 |
| AT | 6 | 3 | 4 | 13 | 0.393 |
| CG | 2 | 0 | 1 | 3 | 0.302 |
|  |  |  |  |  |  |
| AAC | 2 | 9 | 8 | 19 | 0.344 |
| AAG | 0 | 5 | 2 | 7 | 0.107 |
| ACC | 2 | 0 | 1 | 3 | 0.302 |
| AGC | 6 | 0 | 1 | 7 | 0.051 |
| AGG | 0 | 8* | 0 | 8 | <.0001 |
|  |  |  |  |  |  |
| AAAC | 5 | 5 | 2 | 12 | 0.767 |
| AAAG | 13 | 8 | 10 | 31 | 0.255 |
| AAAT | 1 | 33* | 4 | 38 | <.0001 |
| AAGG | 7 | 2 | 7 | 16 | 0.063 |
| ACAG | 3 | 0 | 2 | 5 | 0.164 |
| ACAT | 1 | 1 | 1 | 3 | 1.000 |
| ACGC | 2 | 0 | 0 | 2 | 0.535 |
| AGAT | 9 | 7 | 15 | 31 | 0.128 |
| AGGG | 3 | 3 | 0 | 6 | 0.675 |
| ATCC | 3 | 0 | 0 | 3 | 0.302 |
|  |  |  |  |  |  |
| AAAAC | 3 | 4 | 1 | 8 | 0.475 |
| AAAAT | 0 | 2 | 0 | 2 | 0.135 |
| ACACC | 0 | 0 | 1 | 1 | 1.000 |
|  |  |  |  |  |  |
| Others | 10 | 13 | 9 | 32 | 0.709 |
|  |  |  |  |  |  |
| Total | 294 | 298 | 219 |  |  |
